# Elderly-Onset Rheumatoid Arthritis: Characteristics and Treatment Options

**DOI:** 10.3390/medicina59101878

**Published:** 2023-10-23

**Authors:** Slavica Pavlov-Dolijanovic, Milan Bogojevic, Tatjana Nozica-Radulovic, Goran Radunovic, Natasa Mujovic

**Affiliations:** 1 University of Belgrade, Faculty of Medicine, Institute of Rheumatology, 11000 Belgrade, Serbia; g.radunovic47@gmail.com; 2 Clinical Centre of Montenegro, Department of Rheumatology, 81000 Podgorica, Montenegro; milanbogojevic89@gmail.com; 3 Faculty of Medicine, Institute for Physical Medicine and Rehabilitation and Orthopedic Surgery “Dr. Miroslav Zotovic”, University of Banja Luka, 78000 Banja Luka, Bosnia and Herzegovina; tatjananozica@gmail.com; 4 University of Belgrade, Faculty of Medicine, Center for Physical Medicine and Rehabilitation, University Clinical Centre of Serbia, 11000 Belgrade, Serbia; natashamujovic68@gmail.com

**Keywords:** elderly-onset rheumatoid arthritis, rheumatoid arthritis, prognosis, treatment

## Abstract

Elderly-onset rheumatoid arthritis (EORA) is a distinct clinical entity defined as the onset of rheumatoid arthritis (RA) in individuals aged over 60 years. EORA presents unique clinical features, including a more equitable distribution of sexes, a potential predilection for male involvement, a higher incidence of acute onset characterized by constitutional symptoms, a propensity for systemic manifestations, elevated sedimentation rates at disease onset, a reduced occurrence of rheumatoid factor positivity, increased titers of anti-citrullinated protein antibodies, a preference for involvement of large joints, elevated disease activity, the presence of bone erosions, and heightened patient disability. RA is recognized to consist of three partially overlapping subsets. One subset mirrors the classical RA clinical presentation, while the remaining subsets exhibit either a polymyalgia rheumatica-like phenotype or present with remitting seronegative symmetrical synovitis accompanied by pitting edema syndrome. In the initial stages of EORA management, non-steroidal anti-inflammatory drugs (NSAIDs) are not typically the first-line treatment choice, because seniors are much more prone to develop side effects due to NSAIDs, and the use of NSAIDs is in reality contraindicated to the majority of seniors due to comorbidities. Disease-modifying antirheumatic drugs (DMARDs), frequently methotrexate, are introduced immediately after the diagnosis is made. In cases where elderly patients demonstrate resistance to conventional DMARD therapy, the introduction of biological or targeted synthetic DMARDs becomes a viable treatment option. EORA presents a unique clinical profile, necessitating tailored treatment strategies. Our study emphasizes the challenges of NSAID use in seniors, highlighting the imperative shift toward DMARDs such as methotrexate. Future research should explore personalized DMARD approaches based on disease activity, comorbidities, and safety considerations, aiming to optimize treatment outcomes and minimize glucocorticoid reliance, thereby enhancing the quality of care for EORA patients.

## 1. Introduction

Rheumatoid arthritis (RA) is a systemic, inflammatory disorder that predominantly affects women, with the peak incidence occurring between the ages of 30 and 50, and the average onset age is 55 [1]. Elderly-onset rheumatoid arthritis (EORA) is typically characterized by disease onset after the age of 60. Conversely, “young-onset rheumatoid arthritis” (YORA) describes RA with a more typical presentation at a younger age [1]. Approximately one-third of RA cases develop in individuals aged over 60, and the prevalence increases with advancing age, particularly into the eighth decade [1,2,3]. A recent extensive registry of RA cases in the USA revealed that around one-fourth of enrolled patients received their diagnosis after reaching 60 years of age [4]. In Japan, where the population has aged rapidly in recent years, one major registry study indicated that the peak age at which RA presents has shifted from 50–59 to 60–69 years of age over the past decade [5]. In fact, it is not uncommon in typical Japanese rheumatology practice to encounter patients who develop RA after the age of 70 or even 80. The Japan Gerontological Society previously defined elderly individuals as those over 65 years old, but recently updated this definition to include those over 75 years old [6]. This adjustment holds particular significance due to the global increase in the proportion of older individuals within the population and the potential implications that this has for the care of such patients in the years ahead.

Historically, earlier studies generally concluded that EORA was a milder form of the disease with a favorable prognosis [2,7,8,9]. However, recent research has revealed a more concerning disease activity and severity, as well as poorer clinical, functional, and radiographic outcomes in EORA, compared to YORA [10]. Some authors have postulated that factors such as seropositivity [11,12], longer disease duration [13], and less intensive treatment [9] may contribute to the potentially worse prognosis of EORA. Nevertheless, the fundamental question of whether EORA and YORA genuinely represent distinct prognostic entities or variations within the broader RA spectrum remains unanswered to date.

In the initial stages of EORA management, non-steroidal anti-inflammatory drugs (NSAIDs) are not typically the first-line treatment choice, because seniors are much more prone to develop side effects due to NSAIDs, and the use of NSAIDs is in reality contraindicated to the majority of seniors due to comorbidities. Disease-modifying antirheumatic drugs (DMARDs), frequently methotrexate, are introduced immediately after the diagnosis is made. In cases where elderly patients demonstrate resistance to conventional DMARD therapy, the introduction of biological or targeted synthetic DMARDs becomes a viable treatment option.

In response to the evolving challenges in RA management, the burgeoning field of RA nano therapies holds significant promise. By overcoming the limitations of conventional treatments through advancements in nanotherapeutic techniques, particularly in drug delivery systems, these innovations might offer precise and targeted solutions [14].

EORA presents a unique clinical profile, necessitating tailored treatment strategies. Our study emphasizes the challenges of NSAID use in seniors, highlighting the imperative shift toward disease-modifying antirheumatic drugs (DMARDs) such as methotrexate. Future research should explore personalized DMARD approaches based on disease activity, comorbidities, and safety considerations, aiming to optimize treatment outcomes and minimize glucocorticoid reliance, thereby enhancing the quality of care for EORA patients. 

## 2. Differences between EORA and YORA Diseases

EORA markedly differs from YORA in several key aspects (Table 1).

### 2.1. Sex Distribution

YORA predominantly affects women, with a female-to-male ratio of 3:1. In contrast, EORA exhibits a more equitable sex distribution, with a ratio closer to 1:1 [1,15]. Recent real-world data have even shown a significantly higher proportion of men in the EORA group compared to YORA [10,16,17]. This shift in gender distribution may reflect the intricate interplay between hormone levels (estrogen, progesterone, and androgen) and their influence on the immune system. Notably, RA incidence in women decreases after menopause, when estrogen and progesterone levels decline, while older men with decreasing androgen levels face an increased RA risk [1]. Additionally, one study observed elevated interleukin (IL)-6 secretion associated with dehydroepiandrosterone and androstenedione synthesis in EORA patients, suggesting a potential link between endocrinosescence and immunosenescence [18].

### 2.2. Acute Onset

YORA typically manifests with a gradual onset, characterized by subtle and slow-developing symptoms. Patients in this category often experience a gradual worsening of joint pain and stiffness over an extended period, allowing a relatively gradual progression of the disease. This gradual onset in YORA patients provides a unique perspective into the subtleties of the disease’s early stages, offering crucial insights into the subtle markers and symptoms for which clinicians need to be vigilant.

On the other hand, EORA presents a markedly different clinical picture. Unlike the gradual onset observed in YORA, EORA tends to manifest with a sudden and acute onset, often resembling infectious diseases. Patients in this category might experience a rapid escalation of symptoms, such as severe joint pain, swelling, and fatigue, reminiscent of an acute infection. This acute and infectious-like onset in EORA patients poses unique diagnostic challenges, as it can be easily misinterpreted, leading to delayed diagnosis and treatment initiation.

Exploring these distinct patterns of onset not only sheds light on the heterogeneity of rheumatoid arthritis but also emphasizes the need for a nuanced understanding of the disease across different age groups. Recognizing these differences in onset patterns is essential for early diagnosis, timely intervention, and personalized management strategies, ensuring that patients, whether young or elderly, receive the most effective and tailored care for their specific onset profile [2,4].

### 2.3. Joint Involvement

In classical RA, the hallmark presentation involves the small joints of the hands and feet. However, EORA patients are more likely to experience symptoms in larger, proximal joints, resembling polymyalgia rheumatica [2,4,10]. Notably, recent research by Targońska-Stępniak et al. [16] analyzed 113 consecutive patients (63 EORA and 50 YORA) and found no significant differences in joint involvement at disease onset between the two groups. Both EORA and YORA patients commonly exhibited the involvement of the small joints of the hands, wrists, and knees. In YORA, the small joints of the feet were frequently affected, while in EORA, the shoulders were more commonly involved.

### 2.4. Systemic Manifestations, Sedimentation Rate at Disease Onset, and Changes in Complete Blood Count

#### 2.4.1. Systemic Manifestations and Disease Activity

Both YORA and EORA may exhibit fatigue, weight loss, and an elevated erythrocyte sedimentation rate (ESR) as manifestations. However, these symptoms tend to be more prominent in EORA, suggesting potential differences in etiology, cytokine profiles, or treatment options. Numerous studies have reported lower hemoglobin levels and higher ESR and C-reactive protein (CRP) levels in EORA when compared to YORA [12,16]. Interestingly, elderly RA patients are less likely to develop subcutaneous nodules compared to their younger counterparts [19]. Recent research by Murata et al. [20] demonstrated that EORA patients had higher CRP, ESR, swollen joint counts (SJC), tender joint counts (TJC), and disease activity than YORA patients, as assessed via various disease activity indices, such as Disease Activity Score 28 (DAS28), clinical disease activity index (CDAI), and simplified disease activity index (SDAI). However, these differences tended to diminish at 1 or 2 years after disease onset, with a similar number of patients from both groups achieving low disease activity or remission. Furthermore, a study by Romão et al. [10] performed an ultrasound-guided synovial biopsies prior to conventional immunosuppressive therapy and after 6 months in 140 patients with early RA (<12 months) starting before (YORA, *n* = 99) or after (EORA, *n* = 41) the age of 60. Both before and after treatment, DAS28-ESR was similar, but ultrasound synovial thickening, and power Doppler synovitis, and the Sharp–van der Heijde score were higher in EORA patients. EORA was independently associated with poor treatment response at 6 months and radiographic progression at 12 months. Synovial pathotype, synovitis scores, and cellular infiltration were similar before treatment, but a pauci-immune-fibroid pathotype tended to be more common in YORA at 6 months. Moreover, YORA patients had a marked improvement in all synovitis parameters, whereas EORA presented only mild decreases in synovitis sub-lining macrophage and T cell scores, with no significant changes in lining macrophages, B cells, or plasma cells. They concluded that early EORA presents differently and has a worse overall prognosis than YORA, with poorer clinical, histological, ultrasonographical, and radiographical outcomes.

#### 2.4.2. Platelet Reactivity with Aging

Aging is associated with a decrease in platelet (PLT) counts, particularly in individuals over 70 years of age [21,22]. Research reported that PLT counts remained stable in young-to-middle aged subjects (20–60 years old) but dropped by approximately 10% in older individuals (>70 year of age) [21]. That was probably the reason PLT counts were lower in EORA but comparable with YORA patients, without statistical significance, in the study of Targońska-Stępniak et al. [16]. Although PLT counts decline with age, PLT reactivity generally increases, leading to heightened interactions with other cells and an augmented inflammatory response. This increased PLT hyperactivity is observed in various age-associated conditions, including sepsis, cardiovascular diseases, exaggerated inflammation, and thrombosis [16,21].

#### 2.4.3. Hematological Inflammatory Markers

Novel measures of systemic inflammation, such as platelet-to-lymphocyte (PLR) and neutrophil-to-lymphocyte (NLR) ratios, have been examined in patients with different ages of RA onset (EORA and YORA) in Targońska-Stępniak et al.’s paper [16]. PLR reflects changes in PLT and absolute lymphocyte counts and is regarded as a biomarker for subclinical inflammation, such as cardiovascular disease (CVD), diabetes, sarcopenia, and autoimmune diseases [23,24]. A higher PLR is also associated with poor clinical outcomes in CVD and a poor prognosis in various oncological diseases [24,25]. NLR, predicting poor outcomes in inflammatory disorders [26], rises due to increased ANC and lymphopenia in active RA [16,27]. The NLR cut-off value is 1.4 classified patients in deep remission, with 90% specificity and 24% sensitivity [28]. 

Red blood cell distribution width (RDW) positively correlates with the CRP and ESR of inflammatory and autoimmune diseases [29,30]. RDW appears to be insensitive to infective events and seems to represent long-term inflammatory status, while conventional parameters (CRP, ESR) represent short-term inflammatory statuses [27].

A study by Targońska-Stępniak et al. [16] revealed higher white blood cell counts and ANC in EORA but no lymphopenia and no high PLT counts, resulting in significantly lower PLR and comparable NLR values in EORA compared to YORA patients, possibly indicating a better prognosis. The relevance of PLR and NLR in EORA requires further prospective observation.

### 2.5. Rheumatoid Factor Positivity

Distinct immunoregulatory mechanisms may be at play in the pathogenesis of RA across different age groups. Gamerith et al. [31] observed a significant increase in the ratio of antigen-bound anti-IgG-Fab antibodies (referred to as “hidden aFab”) to free aFab in patients with YORA compared to EORA, leading to an elevated presence of rheumatoid factor (RF). Additionally, some researchers have reported a lower percentage of RF presence in serum at the onset of EORA [17,19], despite the fact that IgM-RF levels tend to increase with age in the general population [32]. However, these findings have not been consistently confirmed by another study [16].

More recently, Murata et al. [20] found no significant differences in the presence of anti-citrullinated protein antibodies (ACPA) or RF between EORA and YORA patients. However, among ACPA-positive patients, EORA exhibited higher ACPA titers compared to YORA. In contrast, Jinno et al. [17], in the ANSWER cohort study, identified a lower percentage of RF positivity in the EORA group, while the percentage of ACPA positivity was similar between the EORA and YORA groups. The variability in these antibody profiles may be another factor contributing to or explaining the distinct clinical courses and prognoses observed across different age groups. Nonetheless, antibody positivity may hold significance as an important prognostic factor in RA.

### 2.6. Anti-Citrullinated Protein Antibodies 

Numerous studies have consistently demonstrated that the risk of radiographic progression is more pronounced in individuals positive for ACPA than in those positive for IgM rheumatoid factor [20,33]. Additionally, EORA has been identified as a risk factor for bone erosions in early RA [20]. This observation aligns with the findings presented by van der Heijde et al. [11], who indicated a trend toward more radiographically detectable damage in older patients compared to younger patients (under 60 years of age) during follow-up. Murata et al. [20] further illustrated that a significantly higher number of EORA patients exhibited bone erosions compared to YORA patients. Consistently, the Larsen score was higher in EORA than in YORA patients. Moreover, EORA continued to have a higher Larsen score at the 2-year mark after onset. Innala et al. [9] also demonstrated a significant association between EORA and the presence of bone erosions, as well as a high Larsen score at both baseline and the 2-year mark.

Recent data indicate that EORA and ACPA positivity are significant risk factors for bone erosion at the 2-year mark in patients who were free from erosion at baseline [20]. Additionally, higher DAS28-ESR at baseline and the presence of EORA are risk factors for bone erosions that remain refractory to treatment [20]. Similarly, Sugihara et al. [8] demonstrated that ACPA positivity, DAS28-ESR at week 0, erosion score at week 0, a lack of response to the European League Against Rheumatism (EULAR) criteria at week 12, and failure to achieve low disease activity at week 24 were prognostic risk factors for radiographical progression at the 1-year mark in EORA.

Study by Murata et al. [20] also indicated that over 25% of ACPA-positive EORA patients exhibited bone erosions at the 2-year mark, even if they achieved remission based on CDAI or SDAI criteria after the 1st or 2nd year. In contrast, less than 10% of YORA patients had bone erosions at the 2-year mark if they attained CDAI or SDAI remission. This suggests that remission according to CDAI or SDAI criteria after the 1st or 2nd year may be insufficient to protect against bone erosions in ACPA-positive EORA patients. Notably, despite equivalent disease activity levels at the 1st or 2nd year after onset, EORA patients used less methotrexate (MTX) and more prednisolone. The use of biological disease-modifying antirheumatic drugs (bDMARDs) at the 1st- or 2nd-year mark was insufficient to completely suppress the incidence of bone erosions in EORA patients. These findings underscore the importance of establishing an optimal target and treatment strategy for preventing radiological damage in EORA patients.

### 2.7. Genetic Predisposition in EORA 

The results of studies investigating genetic predisposition in EORA have been inadequate and, at times, contradictory [34]. Notably, RA-associated DRB1 alleles exhibit variations between early- and late-onset RA, as well as among different ethnic groups. For instance, in a study conducted in Columbia, HLA-DRB1*0403 and *1402 alleles were found to be significantly more frequent in EORA compared to YORA [35]. However, contrasting findings were reported by Hellier et al. [36], who did not find a close association between HLA-DRB1/04-related alleles and EORA when compared to YORA. These discrepancies suggest that the impacts of these genes on susceptibility to disease in EORA may not be particularly significant.

In another study conducted in Spain, a relationship between YORA and DRB1/04 was identified, while EORA was associated with DRB1/01 [37]. Additionally, an increased frequency of DRB1-13/14 was detected in patients with seronegative EORA and polymyalgia rheumatica (PMR). These findings suggest that different cytokines and immune mechanisms may be involved in the pathogenesis of RA between the two age groups.

Overall, the genetic predisposition to EORA remains a complex and evolving area of research, and further investigation is needed to fully understand the underlying genetic factors that contribute to this condition.

### 2.8. Greater Disability of Patients 

The increased functional limitations and severity of joint damage observed in some EORA patients may be attributed to the influence of comorbid diseases commonly seen in the elderly or alterations in the delicate balance between joint damage and repair processes as individuals age [38]. Indeed, the onset of RA in the elderly is considered a poor prognostic factor by several investigators [9,20].

However, it is worth noting that contrary findings have been reported. For instance, Ochi et al. [39] demonstrated that elderly-onset RA had an impact on changes in Health Assessment Questionnaire Disability Index (HAQ-DI scores), indicating functional limitations, but not on changes in CDAI over a 54-week treatment period.

## 3. Clinical Features and Differential Diagnosis

EORA is a heterogeneous disease characterized by three distinct clinical patterns [15]. The most common clinical form (70%) closely resembles classical RA, with positive rheumatoid factor (RF), joint erosions, and a worse prognosis than YORA.

The second form (25%) resembles PMR in its presentation, with the involvement of proximal limb joints. It is typically RF-negative, exhibits an acute onset, does not result in joint erosions, and generally has a favorable prognosis. However, it is important to note that asymmetric nonerosive polyarthritis may occur in 25% of patients with PMR, making a careful differential diagnosis essential. In this context, the presence of anti-CCP positivity in EORA and bilateral subacromial bursitis in PMR can be diagnostic aids. A recent study by Nawata et al. [40] analyzed predictors of EORA with a PMR-like onset in patients initially diagnosed with PMR in 72 patients. During follow-up, 25% of these patients were subsequently diagnosed with RA. Criteria such as RF positivity, semi-quantitative evaluation of synovitis via grey scale ≥ 2, and power Doppler (PD) ≥ 1 in hand joints (wrist, metacarpophalangeal, and proximal interphalangeal joints) were indicative of the potential development of EORA within 1 year of PMR diagnosis.

The third EORA pattern shares the clinical and prognostic similarities of remitting seronegative symmetrical synovitis with pitting edema (RS3PE) syndrome. RS3PE syndrome, first described by McCarty et al. in 1985 [41], is characterized by an elderly onset, an acute onset, symmetrical synovitis and tenosynovitis, pitting edema of the dorsum of the hands and feet, negative RF and ACPA status, the absence of bone erosion, and an excellent prognosis, with low-dose corticosteroid therapy and spontaneous remission within 3–18 months [42,43]. Interestingly, in these subgroup cases, high HLA-B27 positivity has also been reported [15]. To distinguish RS3PE syndrome from EORA, it is essential to assess not only intra-articular but also extra-articular lesions using musculoskeletal ultrasound (MSUS). Patients with RS3PE syndrome typically exhibit more extensive articular synovitis and tenosynovitis, along with more frequent intra-articular synovial effusion, tenosynovitis of digital flexor tendons, and peritendinitis of the digital extensor tendons [44].

Sometimes it is hard to differentiate EORA from PMR and RS3PE (Table 2), but it is important to note that the differential diagnosis of EORA extends beyond PMR and RS3PE syndrome. Other conditions, such as osteoarthritis, spondyloarthropathy, hypertrophic osteoarthropathy, sarcoidosis, connective tissue diseases, systemic vasculitis, paraneoplastic syndromes, crystal arthropathies (such as gout, pseudogout, or chronic pyrophosphate arthropathy), and infectious arthritis (including viral, especially hepatitis C, and bacterial infections, like septic arthritis), should also be considered and ruled out via the diagnostic process.

## 4. Comorbidities

In the elderly, RA often coexists with other chronic medical disorders, such as arterial hypertension, autoimmune thyroid disease, diabetes, ischemic heart disease, metabolic syndrome, cholelithiasis [16], osteoarthritis, osteoporosis, and depression [1]. This coexistence of RA with various other chronic conditions can complicate the management and care of elderly patients with RA, as it requires a comprehensive and multidisciplinary approach to address the diverse health needs of these individuals.

### 4.1. Cardiovascular Risk

Traditional cardiovascular risk factors, inflammatory pathogenesis among inflammatory arthritis and atherosclerosis, and the use of steroids and non-steroidal anti-inflammatory drugs (NSAIDs) contribute to increased cardiovascular risk in RA patients, especially the elderly [45]. Also, metabolic parameters were significantly higher in EORA patients’ body mass index (BMI), serum concentration of creatinine, and uric acid than in those of YORA patients [16].

### 4.2. Risk for Infections

EORA patients often have greater disease activity, leading to early disability and eventually increased immobility, which is a strong risk factor associated with infections such as respiratory and urogenital infections. In addition, the use of immunomodulatory treatment further increases the risk of infection in RA patients [45].

### 4.3. Risk for Developing Malignancies

The immune aging process in RA may contribute to an increased risk of developing malignancies, primarily due to the presence of chronic inflammation and impaired DNA repair mechanisms [45]. Parikh-Patel et al. [46] conducted an assessment of cancer risk among RA patients in California. Their study involved 84,475 RA patients, with a total observation period of 405,540 person-years. During this time frame, 5533 incident cancers were diagnosed among the study’s participants.

The findings of this study revealed several noteworthy patterns in cancer risk among RA patients:

- Lymphohematopoietic cancer: RA patients of both sexes had a significantly higher risk of developing lymphohematopoietic cancer.
- Male-specific risks: Male RA patients exhibited a significantly elevated risk of lung, liver, and esophageal cancer. However, they had a lower risk of prostate cancer.
- Female-specific risks: Female RA patients experienced a notably decreased risk of several cancers, including breast, ovary, uterus, cervix, and melanoma. The magnitude of risk reduction ranged from 15% to 57%, lower than that observed in the general population.

### 4.4. Lung Disease

Lung disease is a prevalent issue among patients with RA, and its occurrence can be attributed to a combination of factors, including the disease itself, comorbid conditions, medications used for treatment, or a combination of these elements. Chronic infections and smoking are recognized as significant contributing factors to lung-related complications in RA patients [45].

Notably, interstitial lung disease (ILD) is more frequently observed in RA patients compared to the general population. The available literature data indicate that the lifetime risk of developing ILD is significantly higher in RA patients, estimated at 7.7%, as opposed to 0.9% in individuals without RA. Several factors were associated with an increased risk of developing ILD in RA patients, including older age at the time of disease onset, male gender, and the presence of more severe RA symptoms. It is important to highlight the substantial impact of ILD on the prognosis of RA patients. Those with RA-associated ILD face a threefold higher risk of mortality compared to RA patients without ILD. Furthermore, the median survival period following an ILD diagnosis is notably short, averaging only 2.6 years. ILD contributes significantly, accounting for approximately 13% of the excess mortality observed in RA patients when compared to the general population [47].

## 5. Treatment

The treatment approach for older patients with rheumatoid arthritis (RA) should align closely with the goals set for younger patients, which include the following issues:

- Controlling disease activity: the primary objective is to effectively manage and control the activity of RA to minimize symptoms and inflammation;
- Preventing structural damage: efforts should be made to prevent joint damage and deformities associated with RA;
- Preserving functionality: maintaining or improving the patient’s ability to carry out daily activities and maintain a good quality of life is a key goal;
- Decreasing excess mortality: RA is associated with an increased risk of mortality, and treatment should aim to reduce this excess mortality [48,49].

It is important to note that the treatment of EORA should not significantly differ from that of YORA [15]. The EULAR recommends that treatment decisions should be based on an assessment of disease activity and consider other patient-related factors, such as comorbidities and safety concerns [48].

### 5.1. Non-Steroidal and Steroid Anti-Inflammatory Drugs

The use of non-steroidal anti-inflammatory drugs (NSAIDs) and corticoids (steroid anti-inflammatory drugs) as adjuncts to RA treatment is a common practice. However, existing studies on this topic are somewhat dated, and there is limited clarity regarding the long-term safety of these medications [50].

In the case of EORA, NSAIDs are often the initial choice of treatment, prescribed to approximately 60% of patients at disease onset, and they were replaced by disease-modifying antirheumatic drugs (DMARDs). The therapy with NSAID was of short duration, and no significant adverse events were associated with NSAIDs [16].

Conversely, a systematic review by Del Grossi Paglia et al. [51] assessed the effectiveness and safety of NSAIDs in treating RA. Out of 10,498 publications reviewed, 26 studies met the inclusion criteria. These studies included 21 focused on NSAIDs (with 10,503 patients) and 5 on corticoids (with 1544 patients). Follow-up time for NSAIDs ranged from 14 to 182 days. The mean age of the patients varied between 46.9 and 58.7 years. The most commonly reported side effects associated with NSAIDs were gastrointestinal events. Regarding corticoids, follow-ups time ranged from 12 to 104 weeks. The mean age of the participants ranged from 39.9 to 58 years. The doses of prednisone 5, 7.5, 10, and 15 mg and prednisolone 7.5 mg administered orally were investigated. Regarding the safety of corticoids, gastrointestinal adverse events were the issues most often reported by patients using these drugs. Corticoids are usually used in association with methotrexate (MTX), and, thus, attention should be focused on the adverse effects of combined therapy. However, this research did not analyze seniors. Seniors have the highest risk for treatment-associated harm, given comorbidity and its treatment [52]. Regrettably, seniors are under-represented or even excluded from clinical trials that provide the evidence base for the treatment of RA [53].

Recently, Ochi et al. [39] examined a cohort of 1750 RA patients, with 59.4% categorized as having EORA and 40.6% as having non-EORA elderly (Y/MORA: younger- and middle-aged onset RA). In the EORA group, there was a higher proportion of glucocorticoid-free patients, but the average glucocorticoid dose was greater in the EORA group compared to the non-EORA elderly group. Similarly, Targońska-Stępniak et al. [16] found that corticoid treatment was more common in YORA patients, typically involving low doses (prednisone ≤ 7.5 mg/day) and often being of a chronic nature.

A recent study by Boers et al. [54] evaluated the effectiveness and safety of adding prednisolone 5 mg/day to the standard care of senior patients with RA (aged 65+). They randomized 451 patients (1:1) to receive prednisolone 5 mg/day (*n* = 224) or placebo (*n* = 225) for 2 years. Patients had a mean age of 72 years, predominantly women, with established severe disease; the mean DAS28 was 4.5. Most patients received treatment for RA and for multiple, often cardiovascular comorbidities: overall, a median of seven different drugs. During the trial, good adherence was found in 89% of prednisolone and 88% of placebo patients. The 63% prednisolone and 61% placebo patients completed the 2-year trial. Discontinuations were similar in both groups for adverse events (both 14%) and active disease (3 vs. 4%); the remainder were mostly due to ‘trial fatigue’ (i.e., reasons related to the trial but not to the studied medication) and COVID-related access issues (19 vs. 21%). The mean time on the studied drug was 19 (SD 8) months. Disease activity was 0.37 points lower on prednisolone, and joint damage progression was 1.7 points lower. The most common adverse events (AE) were mild (41%) or moderate (56%) non-serious infections, without clear differences between the groups. At baseline, about one-third of patients had osteoporosis (history or imaging) but only 13% were treated with antiresorptive drugs. Cotreatment with calcium and vitamin D was instituted in 81% of patients. During the trial, symptomatic and asymptomatic fractures occurred at slightly higher rates in the prednisolone group, but the rate of new compression fractures was not significantly different: prednisolone was 19% versus 15% for placebo, with an adjusted relative risk of 1.27. Over 2 years, spine bone density decreased by about 1% in prednisolone but increased by 3% in placebo patients, resulting in a significant difference. The hip bone density did not change. Other AE was rare without relevant differences, and complaints of ecchymosis, hematoma, and skin atrophy predominantly occurred in the prednisolone group (28 vs. 3 AE). Weight gain was rare, and adrenal insufficiency was not reported. One patient in each group underwent joint replacement surgery. This study adds substantial evidence to support practice rather than guidelines: add-on chronic prednisolone at 5 mg/day for up to 2 years is effective and not particularly dangerous compared to the alternatives. With proper monitoring, prevention and treatment of harmful effects, especially infections and bone loss, titrating around this level will allow the optimum suppression of disease activity.

In current practice, many patients with RA are chronically treated with low-dose steroids [20,55], in direct contradiction to guidelines that prescribe only short-term ‘bridge’ therapy in view of the perceived long-term adverse effects [48]. In these patients, multiple or widespread chronic pain may lead to declined activity and onset of disability. Low doses of glucocorticoids appear to be preferred in such situations [9], but Ochi et al. [39] think that glucocorticoid use could increase the risk of poor treatment outcomes and serious infectious diseases. Therefore, when elderly patients are refractory to conventional therapy, the introduction of bDMARDs or targeted synthetic DMARDs (tsDMARDs) would be a treatment option that can be used to minimize glucocorticoid doses.

### 5.2. Disease-Modifying Antirheumatic Drugs

Disease-modifying antirheumatic drugs used in YORA may also be safely used in the treatment of EORA. However, drug pharmacokinetics and pharmacodynamics in the elderly population are different, while the incidence of different comorbid diseases has increased in this age group, and due to the high number of medications used, caution must be taken in terms of side-effect profile [15]. EORA patients are not always treated with sufficient doses of DMARDs. The literature data on the use of DMARDs in patients with EORA are limited and contradictory.

#### 5.2.1. Hydroxychloroquine/Chloroquine

Since its approval by the U.S. Food and Drug Administration (FDA) in 1955, hydroxychloroquine (HCQ) has found widespread use in the treatment of RA and other rheumatic diseases. HCQ is preferred over chloroquine (CQ) due to its lower toxicity [56]. The potential use of HCQ/CQ for the treatment and prophylaxis of coronavirus disease 2019 (COVID-19) has garnered significant attention, and there is increasing interest in the cardiovascular safety of HCQ [57,58]. Cardiotoxic effects attributed to HCQ encompass a spectrum of issues, including conduction disorders, restrictive cardiomyopathy, left ventricular hypertrophy, ventricular dysfunction, and valvular abnormalities [59]. The association between HCQ and heart failure (HF) has been a topic of debate, with some arguments suggesting a cardioprotective role for HCQ, while others support a potential cardiotoxic effect [58]. In a recent pharmacovigilance analysis, Goldman et al. demonstrated an association between HCQ/CQ use for any indication and HF (reporting odds ratio 2.2). In a meta-analysis that sought to investigate the use of HCQ and CQ in patients with rheumatic conditions such as RA, SLE, and Lupus Nephritis, a reduced risk of cardiovascular disease (CVD) development in HCQ users was reported [60]. Moreover, HCQ treatment in RA patients has been linked to an improved metabolic profile and a decreased incidence of cardiovascular events [61], reduced risk of developing diabetes [62], and reduction in the incidence of chronic kidney disease compared to non-users [63]. Additionally, HCQ therapy has shown a protective benefit for RA patients against non-alcoholic fatty liver disease [64].

Regarding the risk of retinopathy associated with HCQ, it has been characterized as a cumulative dose of 1000 g over a 5–7-year period of use. Considering many EORA patients have eye disease, an ophthalmologist examination is needed in all elderly patients before considering HCQ [65]. In line with these findings, Lin et al. [66] speculated that HCQ treatment could be a protective factor against all-cause mortality in EORA patients. Notably, HCQ treatment appeared to reduce mortality risks in EORA patients with malignancies.

Furthermore, high-dose HCQ has been increasingly employed in clinical studies for anti-cancer therapy and has shown potential efficacy by inhibiting autophagic flux, thereby impeding tumor growth and modulating the tumor immune response [67]. HCQ exerts potent inhibition of Toll-like receptor 9 (TLR9). The overexpression of TLR9 in breast and prostate cancer has been associated with cancer invasion, and the stimulation of the TLR9/nuclear factor kappa B (NF-κB) signaling pathway contributes to gastric cancer migration and proliferation [68,69].

#### 5.2.2. Methotrexate

In general, elderly patients with RA are more likely to receive glucocorticoids and lower doses of MTX compared to their younger counterparts [9,39]. MTX is often chosen as the initial DMARD in approximately 85% of EORA patients, and it is used more often in EORA than in YORA patients [16]. Data from EORA patients in the CORONA database [70] indicated that MTX was slightly more commonly used in EORA patients (2101 patients) than in younger-onset RA (YORA) patients (2101 patients), with rates of 63.9% versus 59.6%, respectively. However, the mean MTX dose was found to be higher in YORA patients. Interestingly, treatment-related toxicity due to MTX was more frequently observed in YORA patients. In contrast, a Japanese study by Murata et al. [20], which focused on RA patients diagnosed between 2011 and 2015, revealed a higher percentage of YORA patients using MTX compared to EORA patients (*p* < 0.001). However, no statistically significant difference was found in the MTX dose prescribed to YORA and EORA patients at 1 year after onset. Notably, the average body weight of patients in this cohort was 56.1 kg, and the MTX dosage per body weight was 0.153 mg/kg/week, equivalent to approximately 8.2 mg/week. It is worth highlighting that the literature suggests that Japanese patients may achieve optimal MTX polyglutamate concentrations at lower MTX dosages compared to patients from Europe or the USA [71]. In this study, prednisolone was prescribed more frequently to EORA patients (*p* < 0.05), which aligns with the existing literature [9,39].

The infrequent use of MTX in elderly patients may be attributed to factors such as declining renal function and low albumin levels, which can contraindicate MTX use. Additionally, EORA patients may have reduced tolerance of MTX due to comorbidities or adverse events. A population-based study in Canada found that increasing age was associated with a greater likelihood of MTX discontinuation in newly diagnosed RA patients [72]. Consequently, bDMARD monotherapy may be a necessary approach for EORA patients. In such cases, interleukin-6 inhibitors (IL-6i) may be a preferable choice over tumor necrosis factor inhibitors (TNFi) due to their efficacy in the absence of concurrent MTX therapy [73].

Recently, Sugihara et al. [74] reported the 3-year outcomes of 197 MTX-naïve EORA patients who were treated using a treat-to-target (T2T) strategy. The mean age of these patients was 74.4 years, with a median symptom duration of 6.0 months. Baseline comorbidities included ILD in 15.7% of patients, chronic obstructive pulmonary disease in 5.6%, chronic kidney disease (calculated creatinine clearance < 60 mL/min) in 57.9%, cardiovascular disease in 15.2%, osteoporosis in 27.4%, and a past history of malignancy in 10.7%. Of the 197 patients, 84.7% initiated MTX at some point during the 3-year observation period, and 41.6% of them received bDMARDs. The MTX dose was increased to a mean of 9.9 mg/week (0.19 mg/kg/week) as the maximum dose within 24 weeks, but 61.1% of patients experienced MTX-associated adverse events. Moreover, 40.2% were unable to reach the maximum approved dose (16 mg/week in Japan) due to renal dysfunction (creatinine clearance < 60 mL/min). The study concluded that escalating MTX to the maximum tolerable dose is a crucial component of the T2T strategy. While an effective dose of MTX was administered, the maximum tolerable dose was low in elderly patients. This lower effective dose of MTX in this Japanese cohort could potentially be explained by racial differences in treatment responsiveness to MTX [70]. Lower achievable MTX doses in EORA patients have also been observed in clinical practice in the USA [70]. Sugihara et al. [74] further concluded that MTX-naïve EORA patients can achieve clinical remission and normal physical function over 3 years by following a T2T strategy, targeting low disease activity (LDA). Adhering to T2T led to better outcomes in elderly patients. Additionally, the T2T intervention using MTX and bDMARDs for EORA had an acceptable safety profile. Physicians will always need to balance the benefits and risks of treatment intensification for elderly patients.

#### 5.2.3. Other Conventional Disease-Modifying Antirheumatic Drugs

Sulfasalazine (SSZ) and other drugs like gold salts were significantly more frequently employed as the first DMARDs in YORA patients than in those with EORA [16]. Studies have indicated that EORA patients often receive initial DMARD monotherapy and glucocorticoids (GC), but less frequently receive combination DMARD therapy with biological drugs compared to YORA patients [15,38]. Additionally, research by Chen et al. [75] demonstrated a lower prevalence of DMARD use in Chinese EORA patients (26.7%) compared to YORA patients (35.4%), particularly for MTX, HCQ, and SSZ.

In Korea, nearly all RA patients are prescribed DMARDs [76]. Among these patients, HCQ was the most commonly used conventional DMARD (73.04%), followed by MTX (57.94%), SSZ (31.16%), leflunomide (LEF) (13.53%), bucillamine (BUC) (9.11%), tacrolimus (2.27%), azathioprine (1.67%), and cyclosporine (1.48%). HCQ was also the more frequently utilized conventional DMARD in EORA patients (74.96%) compared to YORA patients (72.11%), while MTX, SSZ, and azathioprine were more commonly used in YORA patients. LEF showed similar usage rates in both EORA and YORA patient groups. LEF is known to carry health risks in elderly RA patients [77]; however, some studies have reported that LEF is as well tolerated as MTX or SSZ in EORA patients [77,78]. Similar findings have been reported in a previous study [70].

#### 5.2.4. Treatment with Biological/Targeted Synthetic DMARDs in Older Population

Before the advent of bDMARDs and tsDMARDs in recent decades, RA was associated with a significantly higher mortality rate (at least two-fold), particularly in cases of greater clinical severity [79]. However, with the introduction of these newer therapies, the mortality risk for RA patients has substantially decreased [80,81]. It is worth noting that available data from the literature indicate that EORA patients receive bDMARDs less frequently than YORA patients, including both TNFis and other biological drugs [13,16,70,76]. Furthermore, advanced age has been recognized as a significant risk factor for infectious diseases [82] and serious adverse events (SAEs) [83] in RA patients treated with b/tsDMARDs, particularly TNFis [84,85], although some studies have reported no differences [70,86]. This recognition of SAEs has often made physicians cautious about using b/tsDMARDs in elderly patients [9,13,70].

Recent observational studies and post hoc analyses of randomized controlled trials (RCTs) have demonstrated the effectiveness of TNFis [4,87], tocilizumab (TCZ) [86], and abatacept (ABT) [88] in elderly patients. Real-world data suggest that the efficacy of TNFis in older patients is similar to that in younger patients, particularly in terms of clinical response, although functional indexes may differ due to associated comorbidities and the presence of osteoarthritis [89,90]. Notably, results from one study predominantly conducted in a Japanese population showed similar efficacy of TNFis (etanercept, infliximab, or adalimumab) across all age groups [17]. This contrasts with results from larger cohort studies that included patients with long-standing RA [86,90,91,92]. These findings indicate that disease duration may have a greater impact on outcomes than the age of onset, although this conclusion may not be applicable to other patient populations due to differences in patient factors or practice patterns.

Ochi and colleagues [93] demonstrated that the response to TNFis was better in EORA than in YORA in the early stages of biologically naïve RA. However, approximately one-tenth of EORA patients showed little response to a b/tsDMARD, especially TNFis [39]. To improve efficacy and prevent unnecessary adverse events, further research is needed to determine how to identify these non-responders prior to treatment. The reasons for these discrepancies between the two age groups are not entirely clear, but one study reported that EORA patients had higher levels of IL-6 and lower levels of TNF-alpha compared to YORA patients [94]. The acute onset and increased acute phase response seen in EORA may be explained by increased IL-6 levels. Punzi et al. demonstrated elevated IL-6 levels in the synovial fluid of EORA patients compared to YORA patients, while no differences were detected in IL-1 and IL-8 levels [95].

The efficacy of TCZ and rituximab (RTX) may be somewhat lower in elderly RA patients [45]. A French study showed that among 222 RA patients treated with TCZ, those over 65 years old had significantly lower clinical response rates and remission rates compared to younger patients (40% vs. 61% and 28% vs. 46%, respectively; *p* < 0.05) [86]. However, a prospective cohort study using a Japanese RA registry found no significant difference in the rate of severe infections between TCZ and TNFi use (HR 2.23, 95% CI 0.93 to 5.37) [96]. On the other hand, an observational cohort study using data from the British Society for Rheumatology Biologics Register for Rheumatoid Arthritis indicated an increased risk (HR 1.22, 95% CI 1.02 to 1.47) of severe infection with TCZ compared to etanercept, defined as an infection resulting in death, hospitalization, or requiring intravenous antimicrobial therapy [97]. In contrast, another study investigating the safety of IL-6 inhibitors (IL-6i) among elderly RA patients provided additional data suggesting that IL-6i therapy is generally well tolerated among them [98,99]. Patient-specific risk factors such as comorbidities may have a more significant impact on the risk of severe infection than the choice of bDMARDs between TNFi and IL-6i [100].

Data on the effectiveness of RTX in older patients are limited. Another French study analyzed the efficacy of RTX and found that although all age groups showed similar clinical responses, patients aged 65 to 75 years were more likely to respond than those over 75 years during a 1-year follow-up period (OR 3.81, 95% CI 1.14–12.79) [101].

Regarding oral Janus kinase inhibitors (JAKi), namely tofacitinib and baricitinib, available data suggest that their efficacy in older populations is quite similar to that in younger populations [49], but safety considerations differ. Tofacitinib and baricitinib were associated with numerically higher rates of serious infections and herpes zoster infections in elderly RA patients compared to younger patients in clinical controlled trials [102,103]. The summary of product characteristics (SmPC) recommends a reduced dose of 2 mg instead of 4 mg daily for patients aged over 75 years starting on baricitinib. Similarly, filgotinib can be administered at a lower daily dose (100 mg/day) for those aged >75 years compared to younger patients (200 mg/day) [104]. Dose adjustment does not appear to be required for other bDMARDs, but caution is advised when treating elderly RA patients, particularly women over 60 years of age, with infliximab [105]. Furthermore, these DMARDs may offer a valid alternative for patients who already meet the criteria for difficult-to-treat rheumatoid arthritis (D2TRA) due to multiple treatment failures, as they possess mechanisms of action against a greater number of cytokines [105]. The recently published ORAL Surveillance study [106] indicated that the use of tofacitinib in patients over 50 years of age, smokers, and those with at least one cardiovascular risk factor resulted in an increased incidence of adverse events, such as major adverse cardiovascular events and malignancies, compared to TNFi. Consequently, the use of this drug in older populations should be limited. Nevertheless, larger studies are needed before concrete conclusions can be drawn in this regard. In clinical practice, the choice of treatment depends on many nonclinical factors, such as a patient’s cognitive level, access to care, and economic considerations, which may introduce selection bias.

Recently, molecular docking studies become pivotal in discovering new therapies for RA. One in silico study analyzes affinities of known JAK inhibitors and uncovers three promising compounds, highlighting their potential as future treatments [107].

In Korea, adalimumab is the most common bDMARD, followed by etanercept, infliximab, and RTX. While the use of conventional DMARDs does not differ between men and women, interestingly, there is a gender disparity in the prescription of bDMARDs, with women receiving them less frequently than men. This gender difference in healthcare utilization is attributed to women spending less on medical care than men. Additionally, in Korea, women tend to have poorer health status than men but utilize healthcare services less frequently, often accessing lower-quality healthcare services [76].

The Kurama cohort study [20] found that more EORA patients were prescribed ABT or etanercept, while YORA patients were more commonly treated with infliximab. Notably, EORA patients showed significant improvements in DAS28-ESR, SDAI, and CDAI, indicating that EORA patients may have a better response to TNFi than YORA patients, especially in the early stages of RA.

Data from a large Japanese RA registry, which included 7183 RA patients, with 2815 (39.2%) identified as EORA, revealed no significant difference in clinical improvement at 48 weeks between EORA and YORA patients who initiated bDMARDs. However, the proportion of patients on bDMARDs was lower among EORA patients compared to YORA patients (18.3% vs. 28.0%, *p* < 0.001). ABT displayed a favorable benefit profile in elderly RA patients, with the highest retention rate among seven other bDMARDs for clinical response and tolerability. Of the 989 bDMARDs initiators, 364 (36.8%) were identified as EORA. The most often applied bDMARD in EORA was ABT at 29%, followed golimumab (GLM) at 17%, TCZ at 16%, etanercept (ETN) at 13%, certolizumab pegol (CZP) at 10%, adalimumab (ADA) at 6%, IFX infliximab (IFX) at 6%, and JAKi at 3%. In contrast, the most commonly applied bDMARD in YORA was TCZ at 23%, followed ABT at 20%, GLM at 15%, ETN at 13%, ADA at 8%, CZP at 8%, IFX at 7%, and JAKi at 6% [17]. Drug retention and adverse event discontinuation were similar between the EORA and YORA. The sub-analysis showed adverse event rates due to infections were also similar between the two groups [17]. A comparison of treatment use with EORA and YORA is shown in Table 3.

## 6. Adverse Events That Are Related to Drugs

Increased cardiovascular risk has been reported among EORA patients who use glucocorticoids or non-MTX immunosuppressants, such as azathioprine, cyclosporine, or leflunomide [108]. Multivariate logistic regression analysis identified several independent risk factors for EORA complicated with cardiovascular disease (CVD) in Chinese RA patients. These factors include older age (OR = 1.10, 95% CI: 1.00–1.20), a higher number of deformed joints (OR = 3.17, 95% CI: 1.04–9.68), the presence of rheumatoid nodules (OR = 3.56, 95% CI: 1.03–12.23), hypertension (OR = 2.37, 95% CI: 1.09–5.13), and hyperlipidemia (OR = 8.85, 95% CI: 2.50–31.27). In contrast, HCQ (OR = 0.22, 95% CI: 0.07–0.70) and MTX (OR = 0.32, 95% CI: 0.14–0.73) were identified as protective factors against CVD in EORA patients [75].

Previous studies conducted between 1990 and 2007 reported a slight but significant increase in the risk of overall malignancies in RA patients compared to the general population [109]. More recent research by Harigai et al. [110] compared the risk of malignancies in RA patients treated with bDMARDs to the general population. They found that non-hematopoietic malignancies were not significantly elevated in RA patients compared to the Japanese general population, but a significant increase in the incidence of malignant lymphoma was observed. Additionally, a study conducted in Taiwan demonstrated a decreased risk of malignancies in RA patients treated with TNF inhibitors compared to those treated with non-biological DMARDs alone [111]. However, a meta-analysis of Western observational studies showed no significant change in the risk of overall malignancy in patients treated with TNF inhibitors [112]. Reports from Spain and the United Kingdom also indicated no change in cancer mortality rates among RA patients receiving anti-TNF therapy [113,114].

Furthermore, a recent meta-analysis of randomized controlled trials conducted by Lopez-Olivo et al. [115] included data from 63 trials involving 29,423 patients and showed no statistically increased risk of any type of cancer associated with the use of biological response modifiers compared to the control groups. Additionally, a real-world multi-database study found no significant risk of specific cancers associated with various bDMARDs or tsDMARDs in RA patients [116].

## 7. Conclusions

EORA and YORA represent distinct prognostic entities within the spectrum of rheumatoid RA. Despite advancements in science and technology, there are still ongoing controversies in the understanding and management of EORA. Real-world data have revealed that the treatment of elderly RA patients is often unsatisfactory in clinical practice. This can be attributed to several factors, including a lack of robust evidence, concerns about potential adverse events, the presence of comorbidities, polypharmacy, and cognitive dysfunction in elderly patients. As a result, physicians continually face the challenge of striking a delicate balance between the potential benefits and risks associated with treatment intensification in elderly RA patients.

## Figures and Tables

**Table 1:** Comparison of main characteristics of patients with EORA and YORA.

Characteristics	EORA	YORA
Age of onset	after 65 years	30–50
Prevalence	2%	0.5–1%
Female/male ratio	1/1 or more men	3/1
Onset	acute and infectious-like	gradual
Number of joints involved	may be oligoarticular	polyarticular
Sites involved	large/proximal joint	small joints of the hands and feet
Fatigue, weight loss	more prominent	less prominent
Genetic predisposition	HLA-DRB1∗ 01	HLA-DRB1*04
Rheumatoid factor	lower incidence	higher incidence
Titer for ACPA	higher	lower than EORA
Subcutaneous nodules	less frequent	more frequent
Pauci-immune fibroid synovial pathotype	less frequent	more frequent
Bone erosions	more frequent	frequent
Elevated ESR/CRP	more frequent	frequent
Higher WBC, ANC	more frequent	frequent
Lower PLR, NLR	more frequent	frequent
Higher IL6, lower TNFα	more frequent	less frequent
Higher DAS28, CDAI, SDAI	more frequent	frequent
Higher ultrasound features: ST, PDS, SHC	more frequent	frequent
Clinical form	classical RA PMR-like form RS3PE s/m like form	classical RA
Comorbidity	more frequent	less frequent
Prognosis	worse	good or worse

ACPA: anti-citrullinated protein antibodies; ANC: absolute neutrophil count; CDAI: clinical disease activity index; CRP: C-reactive protein; DAS28: 28-joint disease activity score; EORA: elderly-onset rheumatoid arthritis; ESR: sedimentation rate; IL-6: interleukin 6; NLR: neutrophil-to-lymphocyte ratios; SDAI: simplified disease activity index; PDS: power Doppler synovitis; PLR: platelet-to-lymphocyte ratios; PMR: polymyalgia rheumatica; RS3PE: remitting seronegative synovitis with pitting oedema; SHC: Sharp–van der Heijde score; ST: synovial thickening; TNFα: tumor necrosis factor α; WBC: white blood cell count; YORA: young-onset rheumatoid arthritis.

**Table 2:** Different clinical and laboratory features of EORA, PMR, and RS3PE.

Characteristics	EORA	PMR	RS3PE
Proximal joints	+	+++	−
Peripheral joints	+++	+	+++
Tenosynovitis	++	+/−	+++
Edema	+	+	+++
RF+	+/−	−	−
High ESR	+	+++	+

− Absent, + Occasional, ++ Frequent, +++ Very frequent; EORA: elderly-onset rheumatoid arthritis; ESR: sedimentation rate; PMR: polymyalgia rheumatica; RS3PE: remitting seronegative synovitis with pitting oedema.

**Table 3:** Comparison of treatment use with EORA and YORA.

Treatment	EORA	YORA	Reference
NSAIDs	first line and replaced by DMARDs	first line and replaced by DMARDs	[16]
Corticosteroid	first line, higher dose than YORA	as combined drug	[54]
cDMARD:	less frequent, monotherapy	frequent, combination therapy	[15,38]
Hydroxychloroquine	more frequent	frequent	[76]
Methotrexate (MTX)	slightly more common than YORA	mean MTX dose higher than EORA	[9,39]
Sulfasalazine	less frequent	frequent	[16]
Lefunomide	well tolerated	similar	[77,78]
Combination two or more DMARDs	less used	frequent	[15,38]
bDMARDs	less frequent	more frequent	[13,16,70,76]
TNFi	similar effective, in the early stage of bio-naïve RA better response than YORA	similar effective	[93]
Infliximab	caution when treating (women over 60 years old)	no caution when treating	[105]
Tocilizumab	less effective	effective	[45,86]
Abatacept	similar effective	similar effective	[88]
Rituximab	less effective	effective	[45]
Combination DMARD with biological drugs	less frequent	frequent	[13,16]
tsDMARDs-JAKi	similar efficacy, higher rates of serious infections and herpes zoster infections	similar efficacy	[102,103]
Baricitinib	reduced dose to 2 mg daily	standard dose 4 mg daily	[104]
Filgotinib	reduced dose to 100 mg daily	standard dose 200 mg daily	[104]
Drug side effect	more frequent	less frequent	[9,15,16,38,39,45,76,77,78,88,93,102,103,104,105]
MACE and malignancies	more frequent	less frequent	[106]

bDMARDs: biological disease-modifying antirheumatic drugs; cDMARDs: conventional disease-modifying antirheumatic drugs; DMARDs: disease-modifying antirheumatic drugs; EORA: elderly-onset rheumatoid arthritis; JAKi Janus kinase inhibitors; MACE major adverse cardiovascular events; NSAIDs: non-steroidal anti-inflammatory drugs; RA: rheumatoid arthritis; tsDMARDs: targeted synthetic disease-modifying antirheumatic drugs, TNFi: tumor necrosis factor inhibitors; YORA: young-onset rheumatoid arthritis.

## Data Availability

No new data were created or analyzed in this study. Data sharing is not applicable to this article.
